# Mental Treatment for Crime

**Published:** 1920-11-20

**Authors:** 


### MENTAL TREATMENT FOR CRIME.

Under this heading the writer of an article in the daily press discusses the treatment by psychotherapeutic methods of such a condition as kleptomania. He distinguishes this form of conduct from ordinary theft in that it is of a purposeless nature, and compares it with homicidal mania in which a person "suddenly takes up a knife, or some other lethal weapon, and for no reason, and without any definite object of hostility, runs amok with it." The method of treatment to be adopted is by "getting directly at the uncanny subconscious mind, examining it, testing it, and treating it. . . . Our main purpose is to allow the *natural* escape of bottled-up thoughts that are causing damage by their escape in unnatural ways." One can only hope it is all true; but, in spite of the repeated asseverations of enthusiasts, such treatment is still tentative, and the results surely do not warrant dogmatic statements—especially in the lay press. Time enough when a solid mass of facts has been accumulated to announce the efficacy of these innovations in therapeutics. It will be said, however, that this stage has already been reached, and that the efforts to ameliorate or to cure certain mental disorders are more than tentative. That claim has already been made on many occasions in respect of the therapeutic value of visits to shrines, "touching" for king's evil, and so on. Christian Science—or un-Christian nescience as it has been called—has made many similar assumptions. It would be foolish to deny the influence of suggestion in the treatment of some cases of mental disorder; and it appears to matter little how the stimulus is applied. But the danger is in attributing some occult and mystic significance to the means employed.

A fallacy too often overlooked is that because some remedial measure is adopted and the patient improves or recovers, *therefore* our treatment has been efficacious. In so many conditions, too, the disorder is cyclical, or there are remissions of the more acute symptoms. In this way those not fully acquainted with the course of mania-depressive insanity, or of general paralysis of the insane, are apt to be unduly elated when amelioration follows treatment: as a matter of fact there may be no causal connection—except in the lively imagination of the observer! Again, kleptomania or homicidal outbursts may be symptomatic of some grave and progressive nervous disorder—such as, for example, general paralysis of the insane, or in any condition where, acquired inhibition being lost as a result of injury, of tonalmia, or of disease, primitive traits—such as acquisitiveness or impulsiveness—no longer receive the needful checks. Such symptoms may, however, be observed in other conditions where there is not necessarily disease but, perhaps, some excessive physiological change as at adolescence, during pregnancy, or at the climacteric. Often during these phases unusual appetites, waywardness, excessive irritability, lack of modesty, undue eroticism, and many other symptoms may be exhibited for a time, passing off as physiological stability is regained. If psychotherapy can speedily redress the balance so much the better; but, so far as can be seen at present, there is not a complete understanding of the problem to be solved.

